# Single versus double bundle in posterior cruciate ligament (PCL) reconstruction: a meta-analysis

**DOI:** 10.1038/s41598-022-07976-w

**Published:** 2022-03-09

**Authors:** Filippo Migliorini, Andrea Pintore, Filippo Spiezia, Francesco Oliva, Frank Hildebrand, Nicola Maffulli

**Affiliations:** 1 Department of Orthopaedic, Trauma, and Reconstructive Surgery, RWTH Aachen University Hospital, 52074 Aachen, Germany; 2 Department of Orthopaedics, Surgery and Dentistry, University of Salerno, 84081 Baronissi, SA Italy; 3 Department of Orthopedic and Trauma Surgery, Ospedale San Carlo, Potenza, Italy; 4 Barts and the London School of Medicine and Dentistry, Centre for Sports and Exercise Medicine, Mile End Hospital, Queen Mary University of London, London, E1 4DG England; 5 Faculty of Medicine, School of Pharmacy and Bioengineering, Keele University, Thornburrow Drive, Stoke on Trent, England

**Keywords:** Medical research, Signs and symptoms

## Abstract

Posterior cruciate ligament (PCL) reconstruction can be performed using single bundle (SB) and double bundle (DB) techniques. The present study investigated whether DB PCL reconstruction is superior to SB reconstruction in terms of patient reported outcome measures (PROMs) and joint stability. In December 2021 Embase, Google Scholar, Pubmed, Scopus databases were accessed. All clinical trials comparing SB versus DB reconstruction to address PCL insufficiency in skeletally mature patients were considered. Data from 483 procedures were retrieved. The mean follow-up was 31.0 (28.0 to 107.6) months, and the mean timespan between injury and surgery was 11.3 (6 to 37) months. The mean age of the patients was 29.3 ± 3.8 years. 85 of 483 patients (18%) were women. At a mean of 31.0 months post reconstruction, ROM (*P* = 0.03) was slightly greater in the SB group, while the Tegner score (*P* = 0.03) and the Telos stress (*P* = 0.04) were more favorable in the DB cohort. Similarity was found in instrumental laxity (*P* = 0.4) and Lysholm score (*P* = 0.3). The current evidence does not support the use of DB techniques for PCL reconstruction. Both methods could restore knee stability and motion with satisfactory short term patient reported outcome measures. Further high quality clinical trials are required to validate these results on a larger scale.

## Introduction

The posterior cruciate ligament (PCL) restrains posterior tibial translation, preventing external rotation of the tibia^1,2^. The PCL inserts on the intercondylar eminence of the tibia through an anterolateral and a posteromedial bundle^1,2^. These bundles have distinct fibres orientation and tensioning patterns throughout the range of motion of the knee^3–6^. The two bundles synergistically stabilize the knee during the whole range of motion^4,7–9^. The anterolateral bundle is more tense in flexion, and the posteromedial bundle is more tense in extension^10,11^. The anterolateral bundle plays an important role in constraining the mediolateral translation, while the posteromedial bundle controls the anteroposterior translation of the tibia on the femur^7^. An isolated rupture of either bundle does not result in a clinically significant laxity^8,12–14^. On the other hand, when both bundles are injured, the PCL can no longer stabilize the joint, and, if clinical evident instability develops, surgical reconstruction may be indicated^15–19^. Both single bundle (SB) and double bundle (DB) techniques for PCL reconstruction have been described^20–23^. The DB reconstruction technique should more closely replicate the two native bundles of the PCL and the physiological biomechanics of the knee joint. Biomechanically, DB reconstruction better restores the antero-posterior stability than SB techniques^24,25^. However, it is unclear whether DB PCL reconstruction results in better stability and patient reported outcome measures (PROMs) than the SB technique^26–43^. There are no guidelines in support of the number of PCL bundles to reconstruct when undertaking procedure to restore stability. Results from previous meta-analyses and systematic reviews on the topic are inconsistent^44–47^. Recently, clinical studies including a large population which were not been considered in previous reviews have been published^46,48,49^. Increasing pooling data may support clinicians to choose the appropriate treatment for PCL reconstruction. Thus, a meta-analysis was conducted to investigate whether DB PCL reconstruction is superior to the SB technique in terms of patient reported outcome measures (PROMs) and joint stability. We hypothesised that, though DB PCL reconstruction is believed to achieve better knee biomechanics, it does not results in better outcomes following reconstruction of the PCL.

## Material and methods

### Search strategy

This meta-analysis was conducted according to the Preferred Reporting Items for Systematic Reviews and Meta-Analyses: the PRISMA guidelines^50^. The PICO algorithm was followed:

- P (Population): PCL tears;
- I (Intervention): isolated PCL reconstruction;
- C (Comparison): SB versus DB;
- O (Outcomes): PROMs and stability.

### Data source and extraction

Two authors (F.M. and A.P.) independently performed the literature search in December 2021. The PubMed, Embase, Scopus and Google Scholar electronic databases were accessed. The following keywords were used in combination: *knee, posterior cruciate ligament, pcl, reconstruction, arthroscopy, bundle, double, single, strand, clinical outcome, injury, isolated, tendon, hamstring, quadriceps, achilles, tibialis anterior, PROMs, patient reported outcome measures, stability, laxity, complication, instability.* If the title and abstract matched the topic, the full-text of the article was accessed. The bibliographies were screened to identify additional articles. Disagreements were resolved by a third author (N.M.).

### Eligibility criteria

All the clinical studies comparing SB versus DB for PCL reconstruction were accessed. Given the authors language capabilities, articles in English, German, Italian, French and Spanish were eligible. Articles with Level I to IV of evidence, according to Oxford Centre of Evidence-Based Medicine^51^, were considered. Editorials, cohort studies, systematic reviews and meta-analyses, technical notes, narrative reviews, expert opinion and letters were excluded. Animal, biomechanics, and cadaveric studies were also excluded. Articles combining PCL with anterior cruciate ligament (ACL) reconstruction were excluded, as were studies on multiligament injuries. Only studies reporting a minimum of 12 months follow-up were included. Studies involving skeletally immature patients were not eligible. Only articles reporting quantitative data under the outcomes of interest were considered for inclusion.

### Outcomes of interest

Two authors (F.M. and A.P.) independently performed data extraction. The following data were collected: generalities (author, year, type of study), demographic baseline (number of samples, mean age), mean follow-up, time from injury to surgery, type of graft. Data concerning the following outcomes of interest were collected: Lysholm Knee Scoring score, Tegner activity score, visual analogue scale (VAS), IKDC, range of motion (ROM), grade of displacement (Telos stress radiography, KT-1000/2000 arthrometer).

### Methodology quality assessment

The methodological quality assessment was performed by a single author (A.P.) using the Coleman Methodology Score (CMS) The CMS is a reliable and validated tool to evaluate the methodological quality of articles included in systematic reviews and meta-analyses^52^, evaluating the population size, length of follow-up, surgical approach used, study design, description of diagnosis, surgical technique, and rehabilitation. Additionally, outcome criteria assessment and the subject selection process were also evaluated. The quality of the studies is scored between 0 (poor) and 100 (excellent), with values > 60 considered satisfactory.

### Statistical analysis

The statistical analyses were performed by the main author (F.M.). To assess baseline comparability, the unpaired t-test was performed using the IBM SPSS version 25. Values of *P* > 0.05 indicated similarity between the two groups. The meta-analyses were performed using the Editorial Manager Software version 5.3 (The Nordic Cochrane Collaboration, Copenhagen). Continuous data were analyzed using the inverse variance method, with mean difference (MD) effect measure. Dichotomic data were analyzed using the Mantel–Haenszel method and odd ratio (OR) effect measure. The confidence interval was set at 95% in all the comparison. A fixed model effect was set as default. If moderate or high heterogeneity was detected, a random model effect was adopted. Heterogeneity was evaluated through Higgins-I^2^ and $\chi$ ^2^ tests. Values of Higgins-I^2^ were interpreted as low (< 30%), moderate (30% to 60%), high (> 60%). Forest and funnel plot were performed. Values of *P* > 0.05 were considered statistically significant.

### Ethical approval

This study complies with ethical standards.

## Results

### Search result

**Figure 1:**
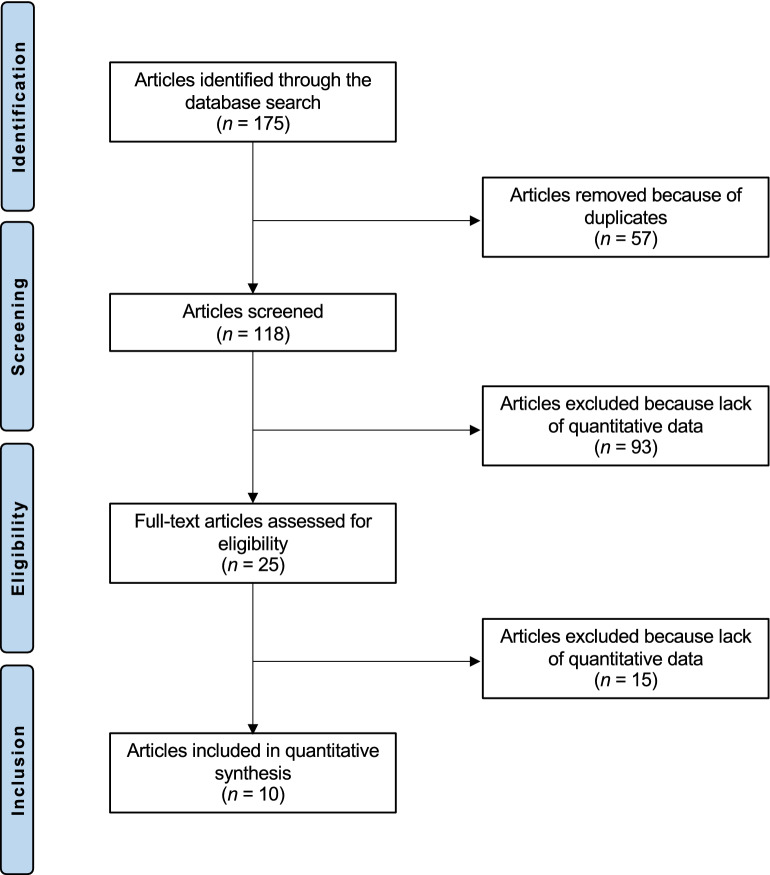
Flow chart of the literature search.

The literature search resulted in 175 articles. After removal of duplicates (N = 57), a further 118 articles were not eligible: language limitations (N = 3), study design (N = 85), combined PCL/ACL reconstruction (N = 7), involving skeletally immature patients (N = 2), short follow-up (N = 6), lacking of quantitative data under the endpoints of interest (N = 15). Finally, 10 comparative clinical studies were included in the present investigation: three randomized controlled trials, one prospective investigation, and six retrospective cohort studies. The literature search results are shown in Fig. 1.

### Methodological quality assessment

According to the CMS, the study size and length of follow-up were adequate. Surgical approach, diagnosis, and rehabilitation were well described in most articles. Outcome measures and timing of assessment were frequently defined, providing moderate reliability. The procedures for assessing outcomes, along with subject selection were often biased and poorly described. Concluding, the CMS scored 49.7 points, attesting the fair quality of the methodological assessment of the articles included in the present meta-analysis (Table 1).

**Table 1:** Coleman methodology score.

Endpoint	Mean	SD	Range
**Part A : Only one score to be given for each of the 7 sections**			
Study size: number of patients	4.8	2.3	0 to 7
Mean follow-up	6.4	3.1	4 to 10
Surgical approach	7.0	0.0	7 to 7
Type of study	4.0	6.6	0 to 15
Description of diagnosis	5.0	0.0	5 to 5
Descriptions of surgical technique	9.0	3.2	0 to 10
Description of postoperative rehabilitation	4.5	1.6	0 to 5
**Part B : Scores may be given for each option in each of the 3 sections**			
Outcome criteria	2.0	0.4	1 to 3
Procedure of assessing outcomes	3.0	0.6	1.5 to 3.5
Description of subject selection process	4.0	2.1	0 to 5

### Risk of publication bias

**Figure 2:**
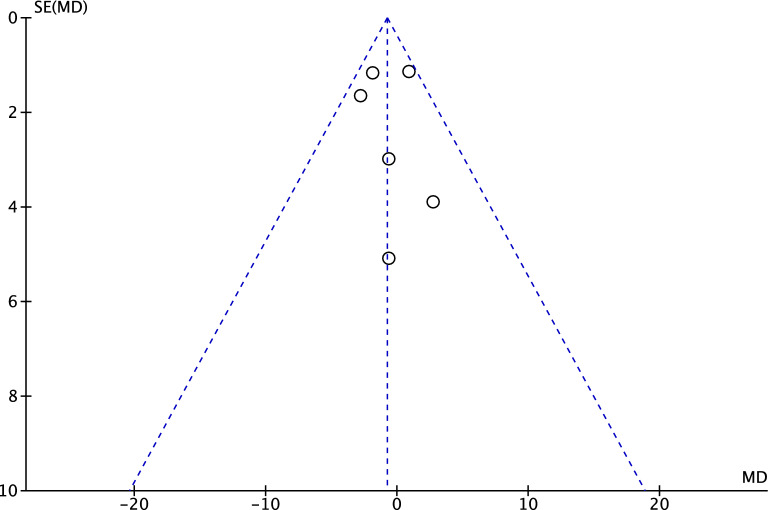
Funnel plot.

The funnel plot of the most commonly reported outcome (Lysholm score) was used to investigate the risk of publication bias. The plot evidenced a very good symmetrical disposition of the referral points. No study was located outside the shapes, increasing the reliability of the plot. Concluding, the risk of publication bias was low (Fig. 2).

### Patient demographics

Data from 483 procedures were retrieved. The mean follow-up was 31.0 ± 49.4 months, and the mean timespan between injury and surgery was 11.3 ± 39.1 months. The mean age of the patients was 29.3 ± 3.8 years. 85 of 483 patients (18%) were women. Good comparability was found at baseline between the two groups in terms of length of follow-up (*P* = 0.9), timespan form injury to surgery (*P* = 0.9), mean age (*P* = 0.4), women (*P* = 0.08), Lysholm scale (*P* = 0.7), ROM (*P* = 0.6), Tegner (*P* = 0.9), Telos stress radiography (*P* = 0.7), arthrometer laxity (*P* = 0.9). Generalities and baseline characteristics of the included studies are shown in Table 2.

**Table 2:** Generalities and baseline characteristics of the included studies.

Author, year	Journal	Design	Bundle	Type of graft	Follow-up (*months*)	Patients (*n*)	Mean age	Female (*%*)
Deie et al. 2015^72^	*Sci World J*	Retrospective	SB	Hamstring	150	27	34.0	33
			DB	Hamstring	150	13	32.0	15
Fanelli et al. 2012^67^	*J Knee Surg*	Retrospective	SB	Tibialis anterior	24 to 72	45		
			DB	Tibialis anterior (PM) & Achilles (AL)	24 to 72	45		
Houe et al. 2004^22^	*Scand J Med Sci Sports*	Retrospective	SB	BPTB	35	16	31.0	50
			DB	Hamstring				
Jain et al. 2016^49^	*Arch Orthop Trauma Surg*	Retrospective	SB	Hamstring	28	22	27.4	0
			DB	Hamstring	28	18	26.4	0
Li et al. 2014^68^	*Arthroscopy*	Randomised	SB	Tibialis anterior	29	22	25.1	32
			DB	Tibialis anterior	30	24	23.5	25
Ma et al. 2019^46^	*Indian J Orthop*	Prospective	SB	Hamstring	28	60	33.6	30
			DB	Achilles	28	30	31.5	27
Shon et al. 2010^73^	*Clin Orthop Surg*	Retrospective	SB	BPTB (71.4%) Achiles (29.6%)	91	14	34.0	18
			DB	Achilles	64	16	36.0	6
Tornese et al. 2008^70^	*Isokinetics Exercise Sci*	Randomised	DB	Hamstring	12	7	24.0	14
			SB	Patellar tendon	12	7	27.0	14
Yoon et al. 2011^69^	*Am J Sports Med*	Randomised	SB	Achilles	31	25	28.5	20
			DB	Achilles	33	28	27.4	11
Yoon et al. 2019^48^	*Am J Sports Med*	Retrospective	SB	Achilles	125	28	29.1	21
			DB	Achilles	131	36	27.0	8

*PM* posteromedial, *AL* anterolateral, *BPTB* bone-patellar-tendon-bone, *SB* single bundle, *DB* double bundle.

### Outcomes of interest

**Figure 3:**
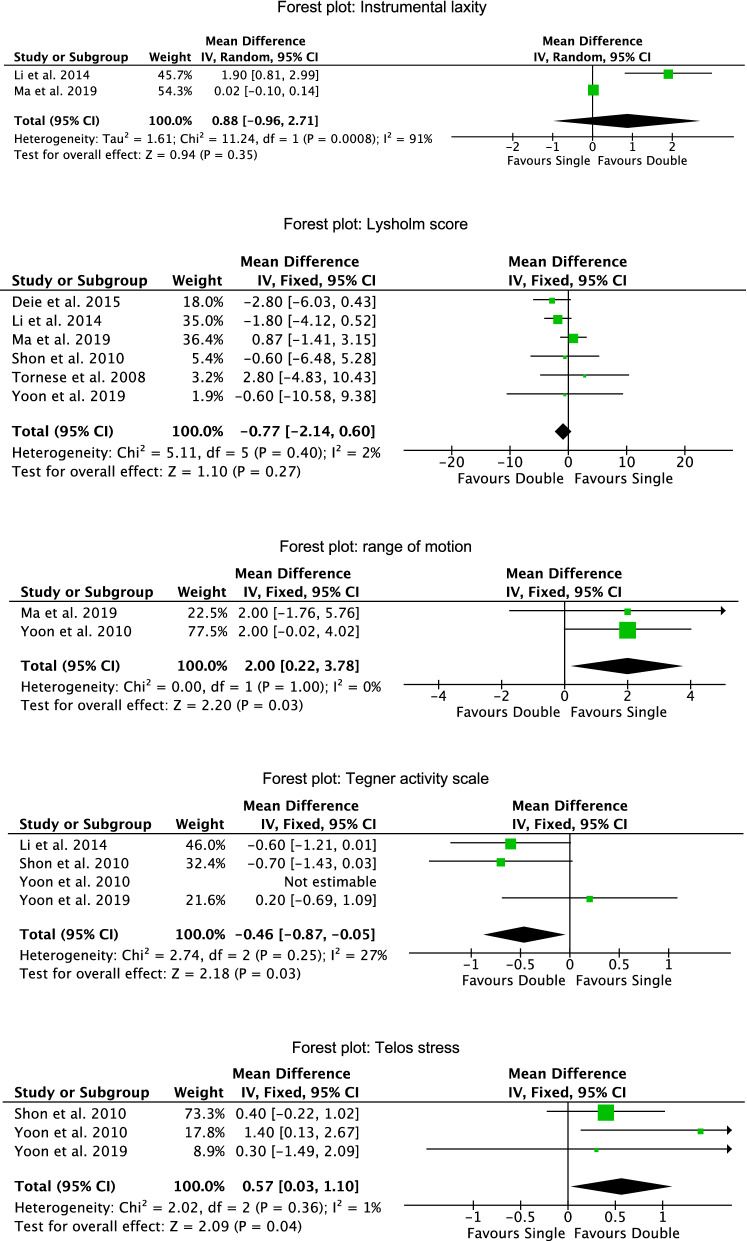
Forest plots of the comparisons.

ROM (MD 2.00; 95% CI 0.22, 3.78; *P* = 0.03) was greater in the SB group, while the Tegner score (MD − 0.46; 95% CI − 0.87, − 0.05; *P* = 0.03) and the Telos stress (MD 0.57; 95% CI 0.03, 1.10; *P* = 0.04), were favorable in the DB cohort. Similarity was found between the techniques in terms of instrumental laxity (MD 0.88; 95% CI − 0.96, 2.71; *P* = 0.4) and Lysholm score (MD − 0.77; 95% CI − 2.14 0.60; *P* = 0.3). The forest plots of each comparison are shown in Fig. 3.

## Discussion

This meta-analysis confirmed our hypothesis of similarity between PCL reconstruction using a SB or a DB graft. DB evidenced minimally greater Telos stress and Tegner score, along with a slightly lower range of motion than the SB. No difference was found in the instrumental laxity and Lysholm score.

The Tegner activity scale is a validated PROM to evaluate the level of activity of the patients^53–55^. Stress radiographs with the Telos stress device are widely employed to evaluate laxity of cruciate ligaments^56–58^. Our results indicated that the Tegner score and the results of the Telos stress were favorable in to the DB cohort; however, the clinical impact of these differences was minimal. Indeed, the MD between the two groups did not overcome the minimum clinically important difference of the Tegner scale, which was estimated between 0.5 and 1 point^59–61^. The instrumental laxity using the AK-1000/2000 and the functional assessment using Lysholm score were also similar, suggesting comparability between the two techniques. Several biomechanical studies stated that DB PCL reconstruction better restore antero-posterior stability than SB techniques^9,12,24,25,62–65^. A recent biomechanical study demonstrated that a DB PCL reconstruction could better restore knee stability across the full ROM, while SB leads to high graft tension during extension and laxity during flexion^66^. Harner et al.^8^ evaluated SB and DB transtibial PCL reconstructions in a cadaveric setting, concluding that DB reconstruction could mimic more closely the physiological knee biomechanics. These results explain partially the findings of the present study. DB reconstruction achieves greater stability according to the Telos stress test, allowing patients to increase their activity level or to quickly return to preinjury level of activity^67^, but also reducing the range of motion. However, the clinical relevance of these findings is questionable, especially in light of the similarity evidenced by the Lysholm score and instrumental laxity. Concluding, even though biomechanical results are encouraging, the clinical outcomes are similar for SB and DB PCL reconstruction. To establish the optimal number of bundles which should be reconstructed, the rate of complications should be investigated. Given the lack of quantitative data concerning the rate of complications experienced by patients after SB and DB PCL reconstruction, no further analyses can be inferred. Only two studies reported data concerning complications after PCL reconstruction surgery^48,49^. Jain et al.^49^ reported four patients with residual laxity and persistent sensation of instability in the SB group; however, only the 5% (1 of 22 patients) underwent revision surgery. Yoon et al.^48^ reported that four patients underwent additional surgeries: one in the SB, and three in DB group. In clinical practice, DB PCL reconstruction present some disadvantages which are worthy of discussion. DB techniques theoretically expose the patients to higher risk of complications, as four drill holes and four fixation devices are required, and pitfalls are possible. Moreover, surgeons must be aware that revision surgery after DB failure may be challenging. Indeed, removal of DB grafts results in bigger bone defects than in SB reconstruction: they can necessitate larger implants for revision, two stage surgeries, higher costs and patient morbidity. Thus, given the similar outcomes, a SB PCL reconstruction may be encouraged as primary choice. It is unclear whether DB should be reserved for revision surgeries.

The retrospective nature of most of the included studies is an important limitation of this eneavour. Unfortunately, only three studies were randomized clinical trials^68–70^, which represents an important source of selection bias. Eligibility criteria and allocation concealment between SB and DB were not clearly stated, and often biased by the studies. The analyses were conducted irrespective of the type of graft used for reconstruction and the tensioning protocol associated with the procedure, representing other important limitations. Instrumental laxity was evaluated regardless to the type of arthrometer (KT-1000 and/or 2000); however, both the instruments provide a static force to the translational displacement of 134 N. The difference between the two instruments is the duration of the test (KT-1000: 2 min and 15 s *versus* KT-2000: 2 min and 3 s), and the methodology of saving the resulting data (KT-1000: manual *versus* KT-2000: digital). Postoperative rehabilitation pattern may also change the biomechanical results, especially at last follow up^71^. However, the rehabilitation process was often biased, and only minimal between-group differences were detectable. Given these limitations, the results from the present study must be interpreted with caution. Finally, further high-quality clinical trials providing long-term follow-up are strongly recommended to establish whether this minimal greater stability affects chondral degeneration, secondary meniscus lesions, onset of osteoarthritis, and to establish the rate of complications of failure of the two bundles.

## Conclusion

Current evidence does not support the use of DB techniques for PCL reconstruction. Both methods could restore knee stability and motion with satisfactory short term patient reported outcome measures. Further high quality clinical trials are required to validate these results on a larger scale.

## Data Availability

The datasets generated during and/or analysed during the current study are available throughout the manuscript.
